# Culturally adapted family intervention for people with schizophrenia in Indonesia (FUSION): a development and feasibility study protocol

**DOI:** 10.1186/s40814-023-01280-8

**Published:** 2023-03-30

**Authors:** Laoise Renwick, Herni Susanti, Helen Brooks, Budi-anna Keliat, Tim Bradshaw, Penny Bee, Karina Lovell

**Affiliations:** 1 Division of Nursing, Midwifery and Social Work, Faculty of Medicine, Biology and Health, School of Health Sciences, University of Manchester, Manchester, M13 9PL UK; 2 Faculty of Nursing, Universitas Indonesia, Depok, Indonesia

**Keywords:** Protocol, Mixed methods, Complex intervention development, Feasibility study, Family interventions for psychosis, Caregiver burden, Co-production, Task-shifting, Low- and middle-income countries

## Abstract

**Background:**

Mental illnesses comprise the single largest source of health-related economic burden globally, and low- and middle-income countries are disproportionately affected. The majority of people with schizophrenia who need treatment do not receive it and are often wholly reliant on family caregivers for daily care and support. Family interventions have an exceptionally robust evidence base for their efficacy in high-resource settings, but it is unknown whether they can produce equivalent effects in some low-resource settings where cultural beliefs, explanatory models of illness and contextual socio-economic issues differ.

**Methods:**

This protocol describes the methods for a randomised controlled trial to determine the feasibility of testing culturally adapt and refine an evidence-based, family intervention for relatives and caregivers of people with schizophrenia in Indonesia. The feasibility and acceptability of implementing our adapted, co-produced intervention via task shifting in primary care settings will be evaluated using the Medical Research Council framework for complex interventions. We will recruit 60 carer-service-user dyads and randomise them in a 1:1 ratio either to receive our manualised intervention or continue to receive treatment as usual. Healthcare workers in primary care settings will be trained to deliver family interventions using our manualised intervention by a family intervention specialist. Participants will complete the ECI, IEQ, KAST and GHQ. Service-user symptom level and relapse status will be measured using the PANSS at baseline, post-intervention and 3 months later by trained researchers. Fidelity to the intervention model will be measured using the FIPAS. Qualitative evaluation will further assist with refining the intervention, evaluating trial processes and evaluating acceptability.

**Discussion:**

National healthcare policy in Indonesia supports the delivery of mental health services in a complex network of primary care centres. This study will provide important information on the feasibility of delivering family interventions for people with schizophrenia via task shifting in primary care settings in Indonesia and allow further refinement of the intervention and trial processes.

## Background

Mental illnesses comprise the single largest source of health-related economic burden globally [1], and low- and middle-income countries are disproportionately affected. Schizophrenia, the most common psychotic illness, is among the ten most disabling conditions worldwide, and global burden is projected to rise [2]. High treatment gaps contribute significantly to illness burden; less than a third of those who develop schizophrenia access treatment in lower resource settings [3]. In Indonesia, as in other lower resource settings, people with schizophrenia are often wholly reliant on family caregivers for daily care and support [4, 5]. Fewer resources, poor infrastructure and a lack of trained professionals to deliver evidence-based care are prominent external factors that lead to increased caregiver burden [6]. Caregivers often attribute supernatural causes to mental illnesses in Indonesia and seek care from traditional healers or shamans [4].

Effective packages of care for schizophrenia comprise both pharmacological and psychosocial interventions [7]. It is broadly considered that, in low- and middle-income countries, a narrower group of interventions will be feasible due to lack of finance and infrastructure, population density and underdeveloped social welfare systems [8]. The World Bank’s recently published third edition of global disease priorities (DCP3) includes family interventions for psychosis as one of only three potentially cost-effective interventions for people with schizophrenia and recommend these interventions should be prioritised in low- and middle-income countries [9]. Family interventions have an exceptionally robust evidence base for their efficacy in high-resource settings [10, 11] and have an emerging evidence base in low- and middle-income countries [12]. Providing these interventions can reduce relapse and improve the family environment and therapeutic alliances with healthcare workers [13].

Family members and caregivers of patients with severe mental illness experience considerable burden [14], exceeding the burden experienced by family members caring for those with comparable long-term physical illnesses such as cancer. Families report significant psychological distress, lower quality of life and increased anxiety and depression [15]. Meta-analytic studies show that interventions comprising both psychoeducation and psychotherapeutic elements can reduce the risk of relapse and rehospitalisation [10, 16], increase adherence to medication regimes [10, 16], enhance functioning [17] and improve family environment [10, 18]. Psychoeducational interventions minimise perceived burden and negative caregiving experiences [11]. There is also evidence that families contribute positively to the wellbeing of people with psychosis, particularly if they themselves are supported by family interventions [16].

However, family interventions designed and tested for delivery in high-income countries may not produce equivalent effects in low- and middle-income countries as they do not allow for cultural beliefs, explanatory models of illness and contextual socio-economic issues to be incorporated into intervention content and delivery [19]. Indeed, when effective interventions are successfully adapted, the acceptability of interventions increases, and people are more likely to engage with help that is offered. Interventions within specific cultural groups delivered in their native language are twice as efficacious as those delivered without adaptation, and cultural adaptation enhances intervention efficacy for treating schizophrenia; the degree of adaptation closely correlated with the degree of efficacy [19].

This study describes a protocol for a mixed-methods inquiry guided by an accepted framework for developing complex interventions to adapt an existing evidence-based intervention. Subsequently, we aim to determine whether it would be feasible to test the effectiveness of this intervention in a randomised trial evaluating recruitment, retention and participant engagement with the intervention. Coinciding with this, we will assess healthcare workers acceptance of the intervention model and optimise sustainability by evaluating barriers and enablers from the perspectives of stakeholders and key informants in positions of potential influence to the delivery of mental healthcare locally and regionally. A third and equal priority aim is to determine whether the intervention can be delivered via shifting this task to non-specialist healthcare workers. Task-shifted interventions for noncommunicable diseases can provide cost savings without compromising on quality [20] and should be explored as an option for lower resource settings to deliver evidence-based treatments and alleviate health system inefficiencies [21].

## Methods

### Aims and objectives

This study details the methods to be used to culturally adapt and refine an evidence-based, family intervention for relatives and caregivers of people with schizophrenia in Indonesia and to evaluate the feasibility and acceptability of implementing these interventions in primary care settings. Using the Medical Research Council framework for complex interventions, we will conduct a three-phase study focusing on earlier phases of development and feasibility testing [22]. We will combine stakeholder consultation, synthesis and consensus workshops using service users, carers and healthcare professional’s perspectives to develop a manual to guide intervention development. We will train healthcare workers to deliver the intervention and assess the feasibility and acceptability of conducting a randomised, single-blind trial of our co-produced, culturally relevant, evidence-based intervention to reduce relapse when compared with standard care.

The study objectives are as follows:

1. Explore preferences and priorities for delivering family interventions for relatives and carers of people with schizophrenia in Java, Indonesia.
2. Synthesise findings from stakeholder interviews with an existing evidence-based intervention using a heuristic model for adaptation.
3. Gain consensus on the components, format and delivery of the intervention.
4. Produce a manual to support the delivery of the culturally adapted intervention.
5. Explore wider factors that may hinder or facilitate the adoption, reach and effectiveness of the intervention delivery and implementation.
6. Train healthcare professionals to deliver the intervention in primary care settings.
7. Assess the feasibility of testing the intervention in a full trial and explore the acceptability of the intervention to a wide group of stakeholders.

The aims of the feasibility trial are to evaluate the following:

1. Acceptability and satisfaction with the intervention
2. Recruitment, attendance and retention in the intervention
3. Completion of outcome measures pre- and post-intervention
4. Recruitment of healthcare workers as therapists
5. Fidelity to the intervention and experience of delivering the intervention

The feasibility aims will be used to inform intervention refinement and development of main trial procedures with respect to intervention acceptability, fidelity and retention in the intervention. Success criteria for progression to a main trial include the following:

1. Recruitment of two dyads/families per centre (three in total) per month
2. Recruitment of a minimum of one healthcare worker per site to deliver the family intervention
3. Completion of 80% of outcome measures at baseline, post-intervention and 3 months later

Success criteria have been reviewed and approved by the Trial Steering Committee.

### Design

Our culturally adapted, co-refined intervention will be tested in a single-blind, randomised trial to determine the feasibility of testing this intervention in a full-scale trial. Carers will be assessed on their level of knowledge, attitudes, burden and general well-being at three time points: pre-intervention, post-intervention and 3 months following intervention completion. Participant dyads in the control group will continue to receive treatment as they usually would; there will be no attempt to withhold any treatment. Additionally, we will conduct individual interviews to obtain qualitative data regarding satisfaction with the intervention and acceptability for delivering the intervention components. This information will be integrated with data from key informant interviews using an implementation framework to develop knowledge about the key facets of intervention delivery by task shifting in primary care settings. Patients and carers were closely involved in the conceptualisation, development and design of this study, and the initial research question was inspired by reports from carer groups of the lack of evidence-based interventions for families of people with schizophrenia in Indonesia. The feasibility trial has been registered (ISRCTN49498363).

### Setting and context

Indonesia is a large archipelago comprising approximately 17,000 islands and roughly 300 different tribes. The prevalence of psychotic illness is 1.8 per 1000, and there is an estimated 2.6 million people with schizophrenia [23]. Mental healthcare is largely provided in one of Indonesia’s large regional public hospitals. Community mental health provision is limited; carers and families receive limited formal support by trained and experienced clinicians, although there is emerging specialist mental healthcare provided within a more advanced network of primary care clinics: puskesmas and posyandu (primary care clinics at district and sub-district level) providing comprehensive healthcare for all healthcare needs at district and sub-district level. Prevailing explanatory models of mental illness favour supernatural theories over biomedical explanations, but often families lack knowledge of treatment availability and approaches to recovery to manage crises and support social functioning. The United Nations Sustainable Development Goals have for the first time focused on reducing the burden of mental illness scaling up prevention and treatment strategies. Coupled with the World Health Organizations focus on task shifting to increase capacity for intervention delivery and integration between primary and secondary mental health services, there is a need to develop family interventions that are evidence-based, can be delivered by non-specialist professionals and are scalable. Task shifting describes when healthcare tasks are redistributed to enhance the performance of health systems. Typically, tasks normally provided by a specialist health worker are transferred to a healthcare worker with a lower level of education and training or a person specifically trained to perform a limited task such as peer or lay workers. We aim to conduct this trial in primary care settings where nonmental health workers are responsible for delivery of mental healthcare due to national healthcare policy in Indonesia which supports the delivery of mental health services in primary care and aims to provide universal healthcare provision for those with diagnosed mental health conditions [24]. Ethical approval has been granted by the University of Manchester Research Ethics Committee for phases 1 and 2 (2020-8041-13687) and phase 3 (2022-14819-25424) of the project.

### Participants and recruitment

Recruitment in the feasibility trial will commence in November 2022. Participants comprising service users, carers and relatives and healthcare professionals through phase will be primarily recruited from primary healthcare centres in Bogor and Jakarta via information posters placed in strategic locations in participating primary care centres. We will also recruit through our NGO partners, Komunitas Peduli Skizofreni Indonesia (KPSI). Our social media strategy for recruitment encompasses public mental health education and awareness groups supported by our NGO partners using varied social media (Facebook, Twitter, Instagram). Inclusion and exclusion criteria are detailed in Table 1.

**Table 1:** Inclusion and exclusion criteria

	*Phase 1*	*Phase 2*	*Phase 3*
*Inclusion criteria*			
*Carers/relatives will be included if they are as follows:*			
Living with or spending at least 10 h per week in face-to-face contact with an individual with schizophrenia and assuming a caring role	✅	✅	✅
Over the age of 18	✅	✅	✅
Resident of Bogor or Jakarta	✅	✅	✅
Able to give informed written consent	✅	✅	✅
*People with schizophrenia will be included if they are as follows:*			
Have a diagnosis of schizophrenia or related psychosis and are currently receiving treatment in a primary care setting	✅	✅	✅
Over the age of 18	✅	✅	✅
Resident of Bogor or Jakarta	✅	✅	✅
Able to give informed written consent as judged by the referring healthcare worker	✅	✅	✅
We will exclude potential participants if they have the following:			
A drug or alcohol dependence alongside a diagnosis of schizophrenia, according to DSM-V criteria	✅	✅	✅
Unstable residential arrangements such that the likelihood of being available for the duration of the trial is low	✅	✅	✅

A sample of 60 dyads including an identified primary carer will be recruited, and these will be allocated randomly to receive either the experimental intervention or treatment as usual aiming for 30 dyads per arm as guided by good practice [25]. Patients and family members will be recruited together and will be asked to contact the researcher directly and separately to confirm participation if they express an interest. Staff in primary care centres will also be given information about the study. Written informed consent will be sought at an initial screening and baseline assessment meeting. Additionally, we will recruit 12–15 healthcare professionals to deliver the intervention by advert and using information giving sessions at primary care centres. Healthcare workers will be included if they have a permanent contract in a primary care centre in Jakarta or Bogor, have primary responsibility for delivering the mental health programme and have delivered family interventions to a minimum of one service-user/family member dyad.

### Intervention development

An existing evidence-based family intervention developed by Barrowclough and Tarrier [26] was adapted using a heuristic framework for culturally adapting psychosocial interventions [19]. We explored stakeholder preferences and priorities for delivering family interviews in consultation groups purposively sampling from service user, carer and healthcare professional groups (6 groups of 8 participants). We conducted two consultation groups per stakeholder group in line with evidence that high levels of both code and meaning saturation can be attained with two focus groups [27]. We explored the views of key informants (*n* = 10) regarding factors affecting implementation of the intervention in primary care settings in individual qualitative interviews. This allowed us to understand the wider implications of intervention implementation and evaluate factors affecting reach, adoption and maintenance of interventions devolved to non-specialist mental health workers in primary care settings. Key informants are individuals who have in-depth knowledge about the community and the target population, community access for healthcare and processes involved in the distribution and delivery of mental health services, and as such are best positioned to identify barriers and facilitators to implementation of interventions and the successful completion of a feasibility study and future trial. Data from consultation groups and individual interviews were analysed using the framework method [28, 29]. Data were analysed by Indonesian researchers, and a sample of scripts (20%) were transcribed and translated, and coding frame agreed with the wider study team at UI and UoM. We devised an evidence matrix combining empirical findings from the qualitative phases of the study with empirical findings from existing evidence synthesis of cultural adaptation for psychosocial and mental health interventions. Guided by the methodology of Lovell et al. [30], the evidence matrix assisted with understanding the commonalities and disagreements between stakeholder views from different sources of evidence and supports decision-making regarding possible content, duration and delivery of the intervention. Areas of divergence were taken forward to a consensus workshop using nominal group technique (NGT), to obtain consensus on aspects of intervention delivery that were not clearly agreed following analysis at the first stage. Originally developed as an organisational planning tool, the NGT has been effectively implemented in mental health research [31, 32] to allow divergent ideas to be expressed and collated with a view to identifying areas of consensus. Each item was presented, and voting conducted in secret, where agreement was not obtained by majority consensus (> 75% concordance); researchers facilitated structured discussion until consensus was reached. The panel was identified and appointed by the RAG, and we determined a sample size of 20 was required to ensure adequate representation from different stakeholders in a mixed-forum event [33].

Our co-produced intervention will be delivered over 10 sessions to individual families comprising the following components: initial assessment of relative and patient needs, education about psychosis, stress management and coping resources, problem-solving and communication therapy and goal setting and relapse prevention. The manual was developed by the wider study team defining therapeutic aims and describing detailed procedures, patient exercises, materials and resources, good practice examples, case studies, skills practice sessions for intervention delivery and measures of processes and outcomes. Consensus was reached regarding the number (up to 10) and duration of sessions (approximately 60 min), location of sessions (domiciliary visit) and including the service user in some elements of the intervention (assessment, giving feedback and education about the illness). Study objectives 1–5 were met during the stages of development of the intervention, and methods for obtaining feasibility objectives (objectives 6 & 7) are described here.

### Outcomes and measures

The primary outcome is to determine the feasibility of family interventions delivered by non-specialist healthcare workers in primary care settings. Feasibility measures comprise evaluations of acceptability, recruitment, retention and fidelity to intervention components and delivery. Secondary outcomes relating to the feasibility of obtaining relevant measures for a full-scale trial comprise assessments of symptom severity and relapse rates, social functioning, family functioning and environment and therapeutic engagement. Analysis will comprise obtaining descriptive statistics and aggregating data to operationalise relapse as an outcome Table 2.

**Table 2:** Variables, measures and assessment points

*Variable*	*Measure*	*Respondent*	*Eligibility*	*Baseline*	*Post-* *intervention*	*Three months post-intervention*	*Mode of administration*
Eligibility	Diagnosis		✅				Case note review
Participant age, gender, relationship to service user, service-user diagnosis, number of hours of contact per week, living status	Demographic measure	Family member	✅	✅			Self-report
Symptom severity	PANSS	Service user		✅	✅	✅	Researcher administered
Relapse rates	PANSS	Service user		✅	✅	✅	Researcher administered
Hospital episodes	Episode count	Service user		✅	✅	✅	Case note review
Social functioning	PSP	Service user		✅	✅	✅	Researcher-administered interview
Family environment and functioning	FQ	Family member		✅	✅	✅	Researcher-administered interview
Knowledge, attitudes, burden and general health	GHQ-28, ECI, KAST, IEQ	Family member, service user		✅	✅	✅	Researcher-administered interview
Therapeutic engagement	Attendance, retention in the intervention	Trial therapist			✅	✅	Researcher-administered interview
Fidelity to the intervention	FIPAS	Trial therapist			✅		Researcher-administered interview
Acceptability of the intervention	Therapist diaries of number of sessions attended	Trial therapist			✅		Self-reported therapist diaries
Satisfaction with the intervention	Qualitative interviews	Family member, service user and trial therapist			✅		Qualitative interviews and self-report questionnaires

#### Psychosis symptom severity and relapse

Clinical symptoms will be evaluated using the PANSS [34] which is a valid scale and has been used in many non-English-speaking countries [35–37]. The validity and reliability of the Indonesian version have also been established [38]. Relapse is operationalised as an increase from mild or below to severe or very severe on one of the following symptoms rated using the PANSS: unusual thought content, hallucinations and conceptual disorganisation for a minimum duration of 1 week [39, 40]. The PANSS will be administered by specially trained research assistants at baseline, immediately after the intervention and 3 months after the intervention. Diagnosis and previous hospital episodes will be obtained from clinical notes, although hospitalisation may be a less relevant proxy measure of relapse in LMIC settings [40].

#### Social functioning

Service-user social functioning will be measured using the Personal and Social Performance Scale (PSP). This is a 100-point, observer-rated, single-item scale comprising occupational activities, relationships, self-care and socially unacceptable behaviours. It is a reliable, acceptable and valid measure of social functioning in people with schizophrenia [41, 42] and sensitive to change in PANSS scores [42] and has been utilised in Indonesian populations previously [43].

#### Caregiver psychological wellbeing

The 12-item General Health Questionnaire (GHQ-12; [44]) was developed to screen for non-specific psychiatric morbidity and has been widely validated and found to be reliable. It is commonly used as a screening tool to determine whether an individual is at risk of developing a psychiatric disorder. It comprises 12 Likert-type question items that measure a single dimension of psychological health. The Indonesian‐language version of the GHQ‐12 has been tested for reliability and validity, demonstrating good consistency and sensitivity (Idaiani & Suhardi, 2006).

#### Caregiver burden

The Involvement Evaluation Questionnaire (IEQ; [45] is a 31-item questionnaire comprising items relating to tension, supervision, worrying and urging and the degree to which the caregiver has experienced any of these. The scale has been developed for European settings, though it has been translated and validated in several European countries and in LMIC settings [46].

Carer burden will also be measured using the Experience of Caregiving Inventory which captures a wider set of negative subscales comprising difficult behaviours, negative symptoms, stigma, problems with services, effects on the family, loss and need for backup. There are two positive subscales consisting of positive personal outcomes and good aspects of the relationship with the patient about the carer’s experiences. This measure has been used with a variety of carers of mental health conditions, and each subscale has been reported to have satisfactory reliability [47].

#### Family functioning and environment

Expressed emotion (EE) will be measured using the Family Questionnaire (FQ; [48]). The questionnaire comprises 20 items each measured on a 4-point scale and consists of two subscales assessing both emotional over-involvement and critical comments. The FQ has excellent psychometric properties including a clear factor structure, good internal consistency of subscales and good inter-rater reliability in relation to the Camberwell Family Interview (CFI; [49]) which is the gold standard measure of EE and is sensitive to predicting components of EE.

#### Knowledge

Knowledge about schizophrenia and psychosis will be measured among participants using the Knowledge About Schizophrenia Test (KAST) [50] which was developed for caregivers of people admitted to hospital for treatment of psychosis. The test comprises 21 items regarding the aetiology, onset, symptomatology, outcome and treatment options. The measure shows excellent content validity and good criterion validity. This scale, the FQ, ECI and IEQ, will be cross-culturally adapted following similar procedures outlined by Knudsen et al. [51], comprising series of translations, back-translations and checking through qualitative inquiry with research assistants and participants in the feasibility study about item validity. Translation will be conducted by researchers independent of the study team, and back-translation will be conducted by the Indonesian researchers in the study team.

### Fidelity to the intervention model

Fidelity to the intervention will be measured using the Family Intervention in Psychosis Adherence Scale (FIPAS) [52]. The scale authors have demonstrated that the majority of items of the FIPAS have acceptable levels of inter-rater reliability. Healthcare workers delivering the intervention will keep a diary following each session to evaluate their opinions of fidelity, factors that they felt may have influenced their fidelity and their views regarding elements that were useful and those that were less useful. Treatment fidelity will also be monitored during qualitative inquiry with healthcare workers who have delivered the intervention. Pre-specified criterion for fidelity will be used interpreting 80–100% adherence as ‘high’ fidelity, 51–79% as ‘moderate’ and 0–50% as ‘low’ fidelity [53].

### Qualitative process evaluation

Qualitative interview data on participants’ views of the intervention will be obtained in individual, semi-structured interviews at intervention completion. These interviews will be digitally recorded, transcribed, checked for accuracy and analysed using framework analysis [54]. Again, data will be analysed by Indonesian researchers, and a sample of scripts (20%) were transcribed, translated and coding frame agreed with the wider study team at UI and UoM. Process evaluation will be informed by the Medical Research Council guidance on process evaluation in designing and testing interventions [55]. We will use a version of the Consolidated Framework for Implementation Research optimised for use in low- and middle-income countries [56] to conduct an implementation analysis that will also inform the feasibility trial which will be finalised prior to a definitive trial utilising qualitative data regarding trial processes integrated with qualitative data collected during the intervention development.

### Procedures

Data will be collected from participants in intervention and control groups at three time points: pre-intervention, post-intervention and 3 months later. Participants within the control group will receive treatment as usual. Standard care comprises limited community services in primary care settings providing public education, counselling, basic psychiatric services [57] and the provision of pharmacological treatment and monitoring at out-patient facilities at regional mental health hospitals. As an active comparator and to evaluate the amount of treatment received by both arms of the trial, all participants will be asked about the extent of treatment and intervention received at regional hospitals and local primary care centres. Demographic data collected include age, gender, ethnicity, marital status, education, and employment will also be collated. As appropriate, we will gather information about the living arrangements, primary diagnosis of service user, duration of the caring role, number of people cared for, relationship to the person with schizophrenia, whether they live with the person, level and type of contact and whether they are receiving support from mental health services. Healthcare professionals will provide information about the nature of their work and whether they have received specific mental health training, contact with people with mental health problems and duration of service Table 3.

**Table 3:**
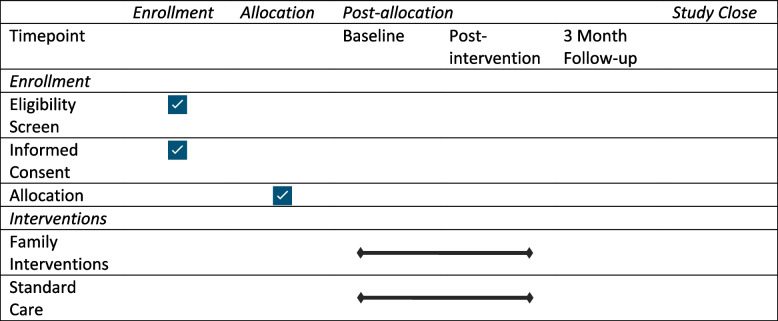
Study enrollment and interventions

### Procedure for randomisation and blinding

Online randomisation services will be provided by a telephone randomisation service (www.sealedenvelope.com), and participants will be allocated in a 1:1 ratio. Randomisation will be conducted according to the International Conference on Harmonization E9 Statistical Principles Guidelines and will be implemented by the trial manager. Upon confirmation of eligibility by the principal investigator in Indonesia (HS), the participant will be randomised to one of the treatment arms. Allocation sequence will be kept from the study researchers responsible for obtaining baseline, and outcome measures and trial management personnel responsible for allocation will work in separate locations. Participants will be asked not to reveal their allocation to the study researchers/study evaluators.

### Training and supervision

Healthcare workers will be trained in the intervention by an experienced, family intervention specialist, and we will train additional academic colleagues who will provide clinical supervision to healthcare workers in practice. The training will be delivered intensively over 1 week comprising 9 workshop sessions with skills practice and an additional session to establish a supervision framework and evaluate the delivery of training. Symptom evaluators will be trained to implement the interview schedule and assess symptoms by trained symptom evaluators among the study team. Symptom evaluators will be blind to treatment allocation, and inter-rater reliability will be measured on concordance between raters on a minimum of 5 standardised observer-rated symptom measures [39, 58]. As above, a supervision framework will be developed during training to ensure that healthcare workers in primary care settings have access to regular supervision with colleagues experienced in mental healthcare and education.

### Research data management

The project will primarily use the Research Data Service at UI to store, manage and curate data. Data will be stored using Word and Excel documents and transferred to research analysis software when required for analysis and distribution checks. Quality checks will be conducted periodically, and coding will be overseen by the study teams at UI and UoM. We will also utilise the UoM Research Data Management Service (RDMS) which provides, managed and secure replicated storage. The RDMS allows researchers to securely transfer digital data to UoM and can be used to store, manage and curate data to preserve this after the lifecycle of the project. Non-digital data, e.g. consent forms and manuals generated from the research programme, will be stored in stand-alone locked cabinets held in a secure location in UI. Data will be stored in raw, processed, analysed and final dataset format to ensure quality and will be transferred between host and sponsor university using Dropbox for Business.

### Trial oversight

The study is supported by a Research Advisory Group (RAG) who are independent of the study team and comprises service users, carers and advocates, healthcare professionals and primary care workers, academics, community leaders and government healthcare officials (*n* = 11). The RAG provide insight and information on the needs of the researchers and the research project using pre-specified terms of reference. The group has provided expertise on research processes and intervention development including comment on analysis of qualitative data, comment on the presentation of intervention and training manuals once developed and comment on assessment schedules for the feasibility study. Additionally, the RAG will provide oversight to the feasibility trial as a Trial Steering Committee chaired by a separate member than oversight of the RAG. The steering committee will approve the final protocol before the feasibility trial commences. The RAG will also take a lead role in the dissemination phase devising plans for investigators and sponsor to communicate trial results to participants, healthcare professionals, the public and other relevant groups. The group will be chaired by the Director of Mental Health and Drug Control, Ministry of Health in Indonesia. Additional service-user and carer representatives will be recruited at each phase and service-user involvement organised by our charity partners Komunitas Peduli Skizofrenia Indonesia (KPSI). KPSI is a user-led charity which runs peer support groups, education and anti-stigma activities in health services and local communities.

## Discussion

This study will provide important information on the feasibility of delivering family interventions for people with schizophrenia who reside in circumscribed areas of Indonesia. The intervention has a solid evidence base for delivery in high-income settings and is a NICE recommended intervention and the model of choice in NHS trusts [26, 59]. Importantly, family interventions have been successfully adapted to minority populations in the UK [60] and in other low-resource settings [61, 62]. As far as we are currently aware, these types of interventions have not previously been adapted culturally to Indonesian populations and contexts nor have co-production methods been utilised in this way to develop an intervention informed by stakeholder perspectives, preferences and priorities, and the findings of this trial will provide a unique and novel contribution to knowledge of family interventions for schizophrenia in this context.

We have focused on implementation within the development phase of this research to ensure that family interventions can be embedded within existing healthcare delivery settings using the more extensive network of primary care services available in Indonesia. Mental health treatment is often provided by mental health professionals whose names and qualifications are maintained in a central government register, and they are licensed to practice in specialty settings. These specialist mental health providers are not readily available in Indonesia due to manpower and resource shortages, fledgling professional development, economically challenged populations and a lack of mental health as a priority in policy agendas. As an example, mental health nurses in Indonesia are not regulated by government legislation, and degree courses have only been available since 2005 [63], and our approach is a unique strength of this study.

Guided by the World Health Organizations promotion of task-shifting models, our aim is to increase capacity for intervention delivery recognising the need for integration between primary and secondary mental health services and to develop family interventions that can be delivered by non-specialist professionals and are scalable [64]. While this is a novel approach in Indonesia, recruitment to our feasibility trial may be challenged by having fewer staff resources to deliver family interventions particularly as we have chosen an individualised intervention. Nonetheless, family interventions delivered intensively have a strong evidence base [10], and our choice partly reflected service-user and carers stated preference for personalised, tailored interventions. Utilising patient-centred approaches and co-design to develop intervention content and processes may also help to overcome challenges of recruiting dyads, which has previously been problematic in family intervention research and practice [17, 65]. To date, we have successfully recruited participants for the first two phases of the project, collected qualitative data and analysed and synthesised findings to inform our culturally adapted manual of evidence-based family interventions. Our coadapted and refined intervention will be administered in this feasibility trial informed by stakeholder preferences and priorities. Our approach aims to enhance the acceptability of and satisfaction with the intervention through embedding relevant and culturally sensitive content and processes within the intervention training and delivery.

Conducting a feasibility study presents multiple opportunities to refine procedures and processes to inform conduct of a more definitive trial at a later point. Indeed, the aim of this trial is to inform the further development of both the intervention and a larger trial with sufficient power to determine the cost and clinical effectiveness of family interventions. We aim to optimise the intervention and trial processes using the oversight of the Trial Steering Committee in determining the feasibility and acceptability of the intervention for a future trial. Our anticipated findings will be a refined, testable, manualised intervention that aims to reduce relapse for people with psychosis and schizophrenia. We will be able to determine whether we can feasibly recruit sufficient participants to test the effects of the intervention and recruit sufficient healthcare workers to determine whether they can be adequately trained to provide such an intervention in its intended format.

## Data Availability

The authors confirm that data sharing is not applicable to this article as no new data were created or analysed in this study.

## Abbreviations

NGO: Non-governmental organisation
KPSI: Komunitas Peduli Skizofrenia Indonesia
TSC: Trial Steering Committee
RAG: Research Advisory Group
RDMS: Research Data Management Service
UI: Universitas Indonesia
UoM: University of Manchester
